# A Countertraction Closed Reduction Technique in Minimally Invasive Fixation of Recent Type C Pelvic Ring Injuries

**DOI:** 10.1111/os.14005

**Published:** 2024-02-22

**Authors:** Wei Liu, Jinmin Zhao, Jianwen Cheng, Linke Huang, Chao Ning, Feng Hu

**Affiliations:** ^1^ Department of Orthopedic Trauma & Hand and Foot Surgery the Second Affiliated Hospital of Guangxi Medical University Nanning China; ^2^ Department of Trauma Surgery the Second Affiliated Hospital of Guangxi Medical University Nanning China; ^3^ Department of Orthopedics Trauma and Hand Surgery The First Affiliated Hospital of Guangxi Medical University Nanning China; ^4^ Department of Bone and Joint Surgery & Sports Medicine the Second Affiliated Hospital of Guangxi Medical University Nanning China

**Keywords:** Closed reduction, Fixation, Unstable pelvic fractures

## Abstract

**Objective:**

Closed reduction of pelvic injuries is a prerequisite and critical step in minimally invasive treatment. Achieving non‐invasive closed reduction of pelvic injuries is a challenging clinical problem. This study demonstrated a non‐invasive traction technique for closed reduction called countertraction closed reduction technique (CCRT) and evaluated its effectiveness for type C pelvic ring injuries.

**Method:**

The data of patients with unstable pelvic fractures treated with CCRT and minimally invasive fixation were retrospectively reviewed from January 2017 to February 2022. Sacroiliac screws were placed to fix the posterior pelvic ring, and internal or external fixation was used to fix the anterior pelvic ring. Operation time, intraoperative blood loss, duration of hospital stay, fracture union and postoperative complications were recorded. Fracture reduction quality was evaluated using the Matta scoring criteria. Functional recovery and general quality of life were evaluated using the Majeed functional scoring criteria.

**Results:**

Thirteen patients (nine males and four females), with an average age of 49.6 years were treated with CCRT and followed up for a mean of 18.5 months. The average operation time was 137.2 minutes (range 92–195 minutes), the average intraoperative blood loss was 31.2 mL (range 10–120 mL) and the average duration of hospital stay was 14.3 days (range 4–32 days). All patients achieved bony union with an average union time of 11.9 weeks (range 10–16 weeks). According to the Matta radiographic criteria, the quality of fracture reduction was excellent in eight patients, good in four, and fair in one. The average Majeed functional score was 89.7 (range 78–100). The functional evaluation revealed that the outcomes were excellent in nine patients, and good in four patients. Complications included incision fat liquefaction in one patient, and heterotopic ossification in another patient. There were no surgical complications as a result of CCRT.

**Conclusion:**

CCRT is a non‐invasive closed reduction method for minimally invasive fixation of fresh Tile C1 and C2 pelvic fractures. The advantages of CCRT combined with minimally invasive treatment include a small surgical incision, reduced intraoperative bleeding, satisfactory fracture reduction, bone healing and functional recovery.

## Graphical abstract

All patients were supine under general anesthesia.The lower limbs of patients in the supine position are placed on the traction, and a pad placed beside the chest or shoulder of the injured side. The uninjured limb was pushed proximally using a traction frame, and then, the injured limb was pulled distally, using the traction frame under fluoroscopic guidance. The final reduction position of the pelvic ring was verified using inlet, outlet, and lateral view radiographs.

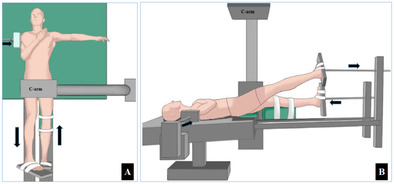

## Introduction

Pelvic fractures are serious injuries with significant morbidity and mortality.^1^ The mortality rate has been reported to range from 7.3% to 14.2% at various times after injury.^2^ The literature has reported that the incidence of pelvic ring fracture increased to 28.3 per 100,000 persons.^3^ The most common cause of pelvic fractures is high‐energy trauma, which includes traffic accidents, crush, and fall injuries.^4^ Studies have shown that complications of pelvic fractures include chronic pain, gait disturbance, post‐traumatic osteoarthritis, and sexual dysfunction.^5^, ^6^, ^7^, ^8^, ^9^, ^10^ Early identification and effective management are crucial to patient survival and functional recovery. The management of unstable pelvic fractures is challenging, and Tile C pelvic injury is a typical unstable pelvic injury characterized by rotational and vertical instability.^11^ In general, the treatment of Tile C pelvic injuries requires fixation of both the anterior and posterior.

Reduction and fixation at the earliest possible stage had the advantage of providing better pain relief and a lower rate of associated complications.^12^ Nevertheless, there is still the possibility of unsatisfactory reduction in open reduction, as huge exposure increases the rate of complications, such as soft tissue injury, bleeding, and risk of infection.^13^ Closed reduction and minimally invasive surgery are alternative treatments that offer significant advantages and decrease the risk of a second hit to the patient.^14^ A minimally invasive surgical technique is extremely useful, particularly with poor soft tissue condition, high risk of infection, and in older adults with chronic medical disease.

It is well known that closed reduction is the first and most important step in minimally invasive treatment for pelvic injury because stability and safety can only be achieved after accurate reduction.^15^, ^16^ While the optimal technique for achieving reduction of the posterior pelvic ring remains controversial. Studies have reported that longitudinal traction is useful for pelvic ring fractures with vertical instability.^17^ The Starr frame,^18^ Matta frame^19^ and unlocking closed reduction technique (URCT)^20^ are useful devices for closed reduction of pelvic fractures. Nonetheless, the lack of hospital infrastructure or doctor training limits the popularization of the frame. When used with closed reduction techniques, achieving anatomical reduction is time consuming, or increases the risk of invasive procedure complications and iatrogenic trauma as reported in previous studies.^21^, ^22^ Few studies^23^ have described the use of an invasive traction table in closed reduction of pelvic ring injuries. Ruatti *et al*.^17^ reported applying strong rapid traction to both transcondylar traction stirrups while two assistants applied manual countertraction to the armpits to reduce isolated sacral fractures.

Based on the above mentioned disadvantages of the patient's conditions, the equipment, and the surgeon, we introduce a closed reduction technique to achieve non‐invasive pelvic reduction procedures, that is the countertraction closed reduction technique (CCRT). The design principle of CCRT is the use of a traction table to pull the affected limb while the unaffected limb resists the traction force, indirectly reducing the fracture and dislocation of the pelvis. This study aimed to evaluate the radiographic and clinical outcomes of the CCRT in the treatment of unstable pelvic fractures based on the following points:

1. What is the clinical effect of treatment of Tile C unstable pelvic ring injuries with CCRT?
2. What are the indications for using CCRT?
3. What are the experience and technical tips of the application of the CCRT?

We hypothesized that CCRT would provide satisfied reduction for minimally invasive fixation of Tile C pelvic injuries.

## Materials and Methods

### Inclusion and Exclusion Criteria

The inclusion criteria were age ≥ 18 years; Tile type C1 and type C2 unstable pelvic ring injuries; reduction with CCRT; external fixation or internal fixation (INFIX) with sacroiliac screws; time from injury to operation of less than 3 weeks. The exclusion criteria were complicated with: (i) diseases affecting fracture healing; (ii) lower limb abnormalities; (iii) patients with head or spinal cord injury affecting functional assessment; and (iv) incomplete follow‐up data or lack of cooperative with treatment.

### General Clinical Data

This retrospective study was approved by the Medical Ethics Committee of the Second Affiliated Hospital of Guangxi Medical University (Number: 2022‐KY[0137]). The study was conducted in accordance with the Helsinki Declaration ethics. A total of 13 patients were identified that fulfilled the inclusion criteria from January 2017 to February 2022. The average age was 49.6 years (range 28 to 74 years). All the patients agreed to participate and provided written informed consent prior to treatment.

There were nine males and four females, with three fractures on the right, five on the left, and five on both sides. The causes of injury were traffic accidents in six cases, a fall from a height in four cases, and a crush injury in three cases. The time from injury to surgery ranged from 2 to 17 days, with a mean of 9.2 days. According to the Tile classification, five cases were Tile C1 fractures, and the other cases were Tile C2 fractures. There were four open pelvic fractures and two Morel‐Lavallée lesions. The baseline data are presented in Table 1.

**TABLE 1:** Demographical characteristics and clinical data of 13 patients.

Variables	(*n* = 13)	Range/percent
Age (years, mean ± SD)	49.6 ± 13.2	28–74
Male, *n*	9	69.2%
Fracture side, *n*		
Right	3	23.1%
Left	5	38.5%
Both sides	5	38.5%
Injury type, *n*		
Traffic accident	6	46.2%
Fall from height	4	30.8%
Crush injury	3	23.1%
Tile classification, *n*		
Type C1	5	38.5%
Type C2	8	61.5%
Open fracture	4	30.8%
Morel‐Lavallée lesions	2	15.4%
Time to surgery (days, mean ± SD)	9.2 ± 5.1	2–17

Classification of pelvic injuries is critical for satisfactory reduction of the pelvic fracture. Pelvic fractures types were classified, based on radiological data, by two orthopedic surgeons (FH and CN). The third senior orthopedic surgeon (JZ) was invited to discuss if disagreements arose. Radiographs and computed tomography (CT) plain scans are important imaging data for the classification of pelvic injuries. Three‐dimensional reconstructions can provide overall information about a pelvic injury. CCRT is best indicated for Tile C1 pelvic fractures and should be used cautiously with Tile C2 pelvic fractures, and is contraindicated for Tile C3 pelvic fractures.

### Surgical Technique

#### CCRT Inspiration and Principles

**FIGURE 1:**
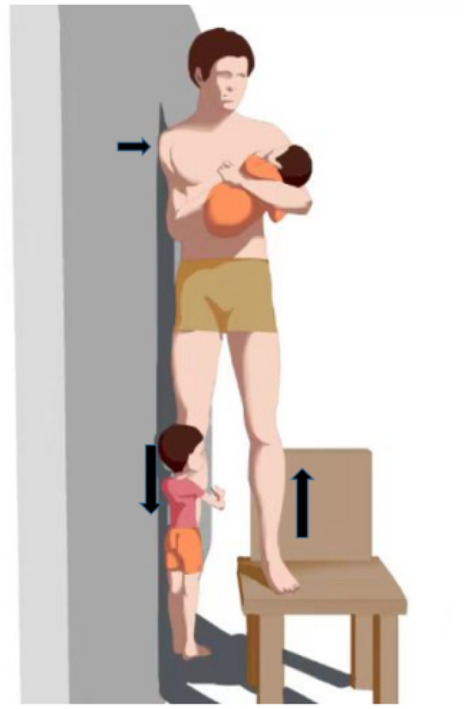
Schematic of video. The man's right shoulder reclines against the wall and carries his baby with his left hand. Meanwhile, he swings the right hip joint, like a rocking chair, to amuse his daughter.

The mechanism of CCRT is to use a traction table to distract the injured lower limb, while the uninjured lower limb acts as countertraction to complete the pelvic reduction under fluoroscopy monitoring. Briefly, the affected limb plays the role of traction and reduction, while the unaffected limb maintains the position of the pelvis. Gradually adjusting the traction force and orientation of the injured limb under the guidance of the image results in the reduction of pelvic fractures and dislocations. The mechanism was inspired by a picture of a man standing on a stool with his left foot, while his daughter was sitting on his suspended right foot (Figure 1). The reaction force of the man's left leg pushes the right leg towards the pelvis in the proximal direction, while the child's gravity pulls the right leg distally towards the pelvis.

### Preoperative Preparation

The lower limbs of patients in the supine position are placed on the traction, and a pad placed beside the chest or shoulder of the injured side, with the purpose of blocking lateral displacement, followed by pushing the unaffected lower limb proximally, forming a countertraction effect which resisting the force of traction of the injured side, and prevent the longitudinal sliding when the patient under the traction force.

**FIGURE 2:**
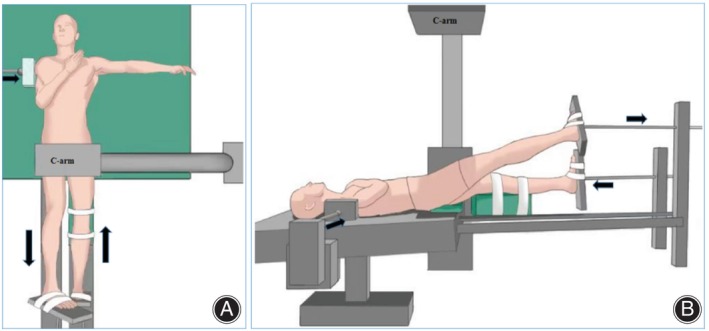
The mechanism of the countertraction closed reduction technique (A, top view; B, lateralis view). The uninjured left of the lower extremity was pushed to resist the longitudinal shift of the body and the pad prevented lateral displacement during the injured right lower extremity traction. The reduction of the pelvic ring injuries was performed by manipulating the traction under image guidance.

All patients were under general anesthesia and the traction table was connected to the surgery table. The patient was positioned under an image intensifier that was previously prepared for free mobilization in the anteroposterior, lateral, and Judet views. A hip positioning pad was applied to prevent lateral displacement of the trunk. Folded surgical drapes were placed under the lumbosacral region to allow access for sacroiliac screw placement. No perineal posts were used in this study. Both the lower limbs were placed in the traction table. Bandages and drapes were used to maintain extension of the uninjured hip and knee. The head‐down and foot‐high positions were selected by setting the tilt of the operating table (Figure 2).

### Reduction and Implant Application

All the operations were performed by an experienced orthopedic surgeon (WL), who has been practicing for more than 10 years. First, the injured lower limb was adjusted to 60° of internal rotation, 10° of adduction, and 15° of hip flexion. The unaffected lower limb was placed in a neutral or 3° to 5° abduction position. Subsequently, the uninjured limb was pushed proximally using a traction frame, until the knee joint was slightly tense. The injured limb was pulled distally, using the traction frame under fluoroscopic guidance, until the posterior pelvic ring returned to an anatomical arc under fluoroscopy in the pelvic inlet view. It showed that the pelvis recovered its annular on the inlet view, indicating that the posterior displacement of the fracture was reduced. Then, the vertical displacement of the pelvis was checked on the outlet view. If the superior pubic ramus of the injured side was higher than that of the contralateral side, then reduction of the flexion angle of the hip joint was indicated; otherwise the flexion angle was increased. Pronation and adduction of the lower extremities were beneficial for reduction of pelvic displacement. Bone reduction forceps were sometimes required to assist in percutaneous reduction of the symphysis pubis dislocation and in maintenance of its reduction. The final reduction position of the pelvic ring was verified using inlet, outlet, and lateral view radiographs. Once the fractures had been anatomically reduced, two guidewires were placed under fluoroscopic control in lateral, inlet, and outlet views, as described by Routt *et al*.^24^ The anterior pelvic ring was fixed with an external fixator or INFIX before placing the sacroiliac screw. Finally, partially threaded iliosacral screws, 7.3 mm (Howmedica, Rutherford, NJ, USA) were used to fix the posterior pelvic ring.

All temporary fixation devices, such as forceps, Kirschner wire were removed and then the traction was loosened. Saline was used to flush the incision and a standard closure of the layers was performed.

### Postoperative Protocol

All patients received 10 mg of rivaroxaban orally daily for prevention of deep vein thrombosis and were given intravenous antibiotics to prevent infection. Plain AP pelvic radiographs and CT scans were performed postoperatively. Early functional exercises without weight‐bearing were performed 2 days postoperatively. Touch weight‐bearing was performed at 4 weeks and gradual full weight‐bearing at 6 weeks. The wound was disinfected and the dressings were changed every 2 days and the incision sutures were removed 2 weeks postoperatively.

All patients were advised to return to the outpatient clinic at 1, 2, 3, 6, and 12 months after surgery to evaluate fracture healing and postoperative complications. The Majeed functional score was assessed at the last follow‐up and a researcher (JC) who was not involved in the diagnosis, treatment, and follow‐up of patients, evaluated the quality of fracture reduction using the Matta score.

### Observational Indicators

Operation‐related indices included surgical procedures (posterior and anterior fixation), operation time, blood loss, duration of hospital stay, and postoperative complications. The clinical outcomes included the pelvic fracture reduction quality, fracture union rate, mean union time, and functional evaluation results. The Matta score was utilized to evaluate the pelvic fracture reduction quality.^25^ Fracture union was defined as the presence of bridging callus on three views of pelvic X‐rays. Pelvis functionality was evaluated with the Majeed score.^26^

### Statistical Analysis

SPSS 21.0 (IBM, Armonk, NY, USA) was used to perform the statistical analysis. Descriptive data were expressed as means ± standard deviations (SD), medians and interquartile ranges (IQR), exact values (n), percentage (%), and range.

## Results

### General Results

The average follow‐up time was 18.5 months (range 12 to 36 months). After CCRT reduction, the posterior pelvic ring was fixed with a sacroiliac screw in all patients, and the anterior ring was fixed with INFIX in nine cases, while the others were fixed with an external fixator (Table 2). The average operation time was 137.2 min (range 92 to 195 min). The average intraoperative blood loss was 31.2 mL (range 10 to 120 mL). The average duration of hospital stay was 14.3 days (range 4 to 32 days).

**TABLE 2:** Operation data, clinical outcomes, and postoperative complications of 13 patients.

Variables	(*n* = 13)	Range/percent
Radiographic union, *n*	13	100%
Union time, weeks, mean ± SD	11.9 ± 1.8	10–16
Surgical procedures		
SISF + INFIX	9	69.2%
SISF + EXFIX	4	30.8%
Operation time, min, mean ± SD	137.2 ± 28.1	92–195
Blood loss, mL, median	31.2 (13–35)	10–120
Hospitalization time, days, mean ± SD	14.3 ± 7.5	4–32
Postoperative complications, *n*		
Incision fat liquefaction	1	7.7%
Heterotopic ossification	1	7.7%
Matta score, *n*		
Excellent, *n*	8	61.5%
Good, *n*	4	30.8%
Fair, *n*	1	7.7%
Poor, *n*	0	0
Majeed score, mean ± SD	89.7 ± 7.8	78–100
Excellent	9	69.2%
Good	4	30.8%
Fair	0	0
Poor	0	0
Follow‐up time (months, mean ± SD)	18.5 ± 6.3	12–36

Abbreviations: EXFIX, external fixation; INFIX, subcutaneous internal fixation; IQR, interquartile range; SISF, sacroiliac screws fixation.

### Radiographic and Functional Evaluation

**FIGURE 3:**
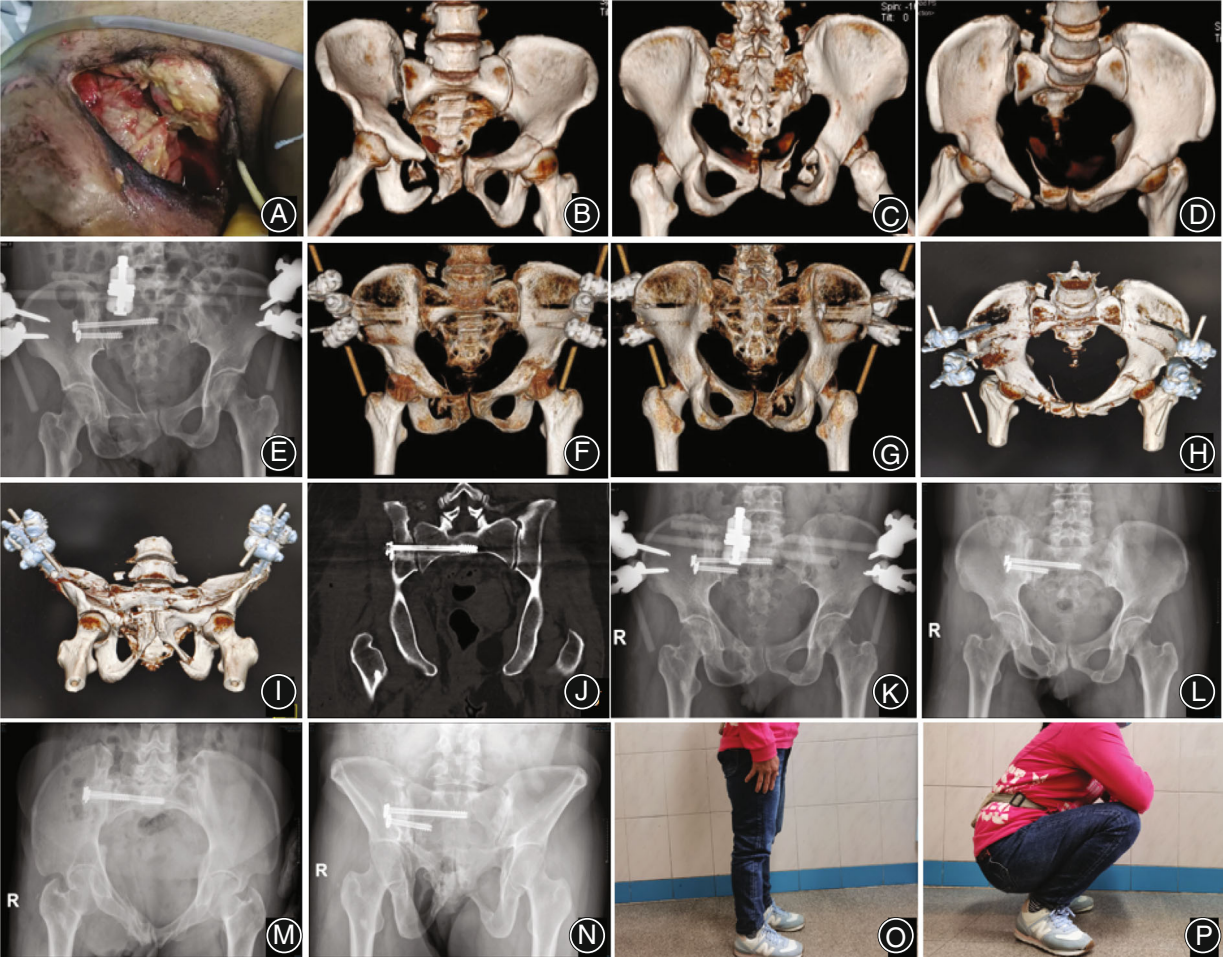
A 43‐year‐old woman with pelvic ring injures (Case 1) caused by a traffic accident. (A) Open injury of right perineum. (B, C, D) Preoperative three‐dimensional computed tomography image showing sacroiliac joint dislocation and bilateral pubic ramus fractures. (E–J) Two days postoperative plain X‐ray film and three‐dimensional computed tomography images showed satisfactory reduction after countertraction closed reduction technique and the fixator was in the right position. (K–N) Three‐month postoperative plain X‐ray radiographs showing healed fractures and the external fixator was removed. (O, P) Follow‐up 3 years after surgery showed good function.

**FIGURE 4:**
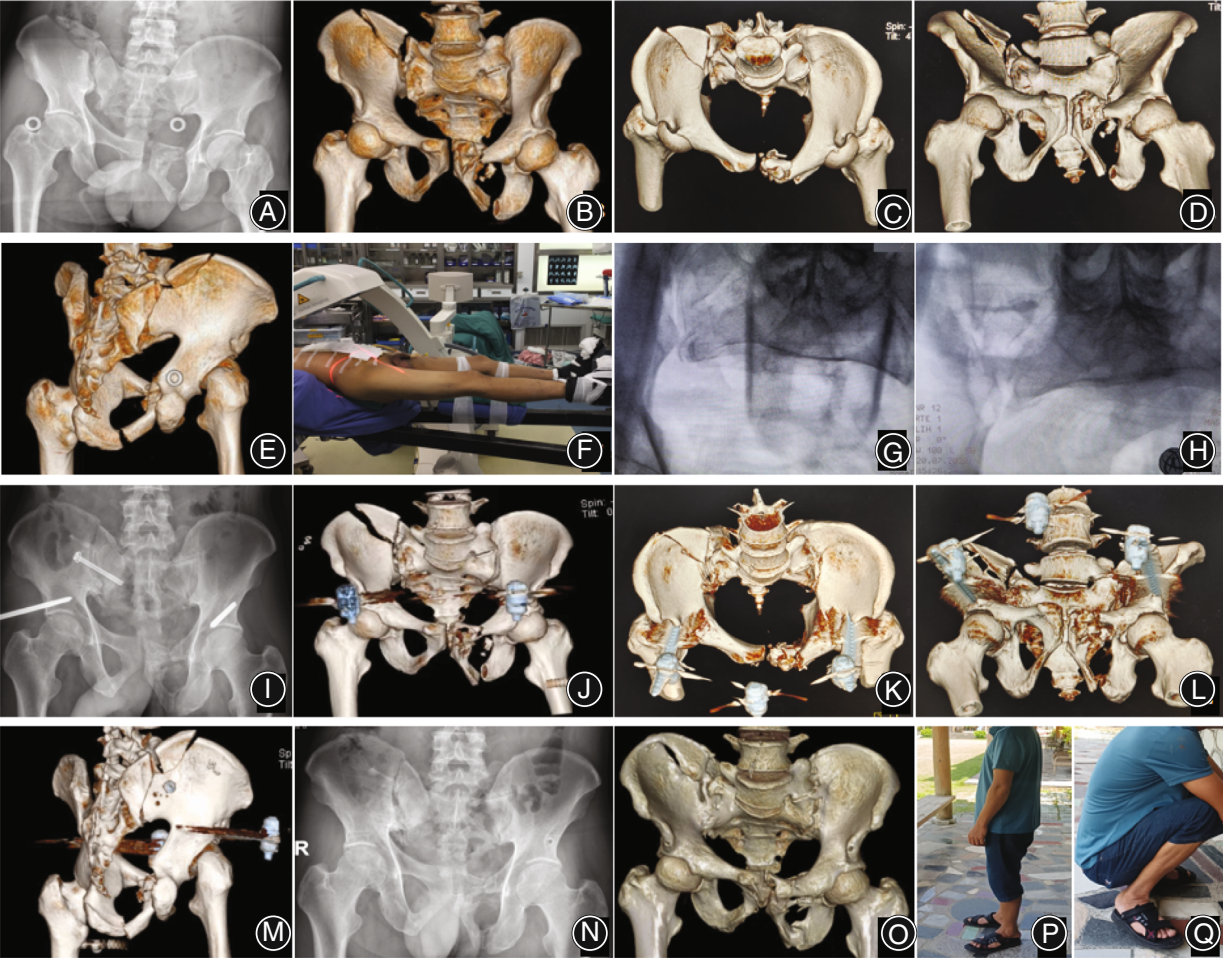
A 42‐year‐old man with pelvic ring injuries (Case 2) caused by a heavy crush injury. (A–E) Preoperative plain x‐ray radiographs and three‐dimensional computed tomography images showing a right iliac fracture with sacroiliac joint dislocation and left pubic ramus fractures (F–H) The countertraction closed reduction technique to reduce the fractures. (I–M) Two days postoperative plain X‐ray radiograph and three‐dimensional computed tomography images showing good reduction of the pelvic ring. (N, O) Plain radiographs and three‐dimensional computed tomographic images three‐months postoperatively showing healing of the pelvic fracture and the external fixator was removed. (P, Q) Follow‐up 2 years after surgery showed excellent function.

**FIGURE 5:**
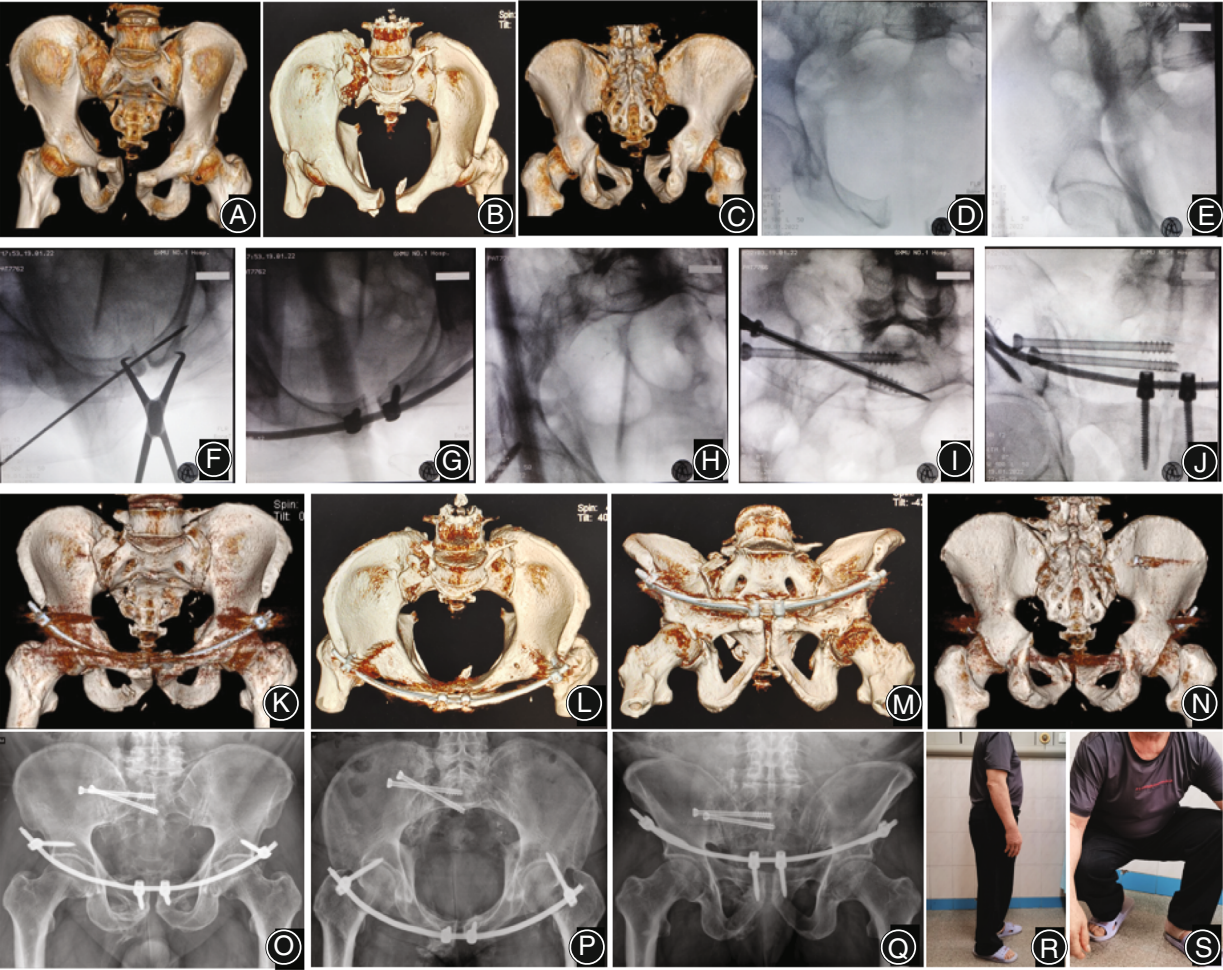
A 72‐year‐old man with pelvic ring injuries (Case 3) caused by falling from a height. (A, B, C) a preoperative three‐dimensional computed tomography image showing a vertical sacral fracture and dislocation of the symphysis pubis. (D, E, F, G, H, I, J) intraoperative imaging of fracture closed reduction and fixation. (K, L, M, N) two days postoperative three‐dimensional computed tomography image showing good reduction. (O, P, Q) plain radiograph image at six‐months postoperatively showing healing of the pelvic fracture and no loosening or fracture of the internal fixation. (R, S) follow‐up 1 year after surgery showing excellent function.

Postoperative CT scans confirmed that the sacroiliac screws were in a safe area and that no screws had inadvertently injured nerves and vessels. Meanwhile, we reviewed coronal and sagittal postoperative CT scans for residual displacement. Using the described reduction technique, we found that 92% (12/13) of patients were considered excellent and good according to the Matta scoring criteria. Only one case was rated fair because the X‐ray machine broke down during the process. The average Majeed functional score was 89.7 (range 78 to 100), and was excellent in nine patients and good in four patients at the last follow‐up. Typical cases are shown in Figures 3, 4, 5.

### Complications

Fat liquefaction necrosis occurred in the incision of one patient; no bacteria were found in the culture of the incision secretion and the wound healed after a dressing change. One patient with dislocation of the pubic symphysis developed heterotopic ossification adjacent to the superior and inferior branches of the pubic, but it did not cause discomfort and was not further treated. No surgical complications occurred as a result of CCRT.

## Discussion

### Clinical Effects of the CCRT

The effectiveness of CCRT in treating unstable pelvic ring injuries is investigated in 13 patients. The clinical data showed that CCRT can achieve non‐invasive reduction efficacy and provide a cornerstone for minimally invasive internal fixation treatment. All of the patient's fractures healed and with satisfactory function. There were no complications associated with CCRT.

### Indications of CCRT

CCRT was suitable for fresh Tile C1 and C2 pelvic fractures because these two types of pelvic injuries preserve the vertical stability of the posterior ring on one side of the pelvis, and the sacroiliac ligament can play the role of the countertraction structure. Interestingly, the type of superior pubic ramus fracture in the first case in this study was a Nakatani type III fracture, which involved the anterior wall of the acetabulum but did not affect the process of CCRT. Analysis showed that the reasons for this were the stability of the posterior pelvic ring ligament and the normal iliofemoral arch.

In the past, surgery delayed for more than 5 days after injury was associated with a poorer quality of closed reduction.^27^ However, with the development of surgical techniques and improved instruments, excellent reduction of pelvic fractures beyond 10 days can still be achieved by closed reduction.^28^ In this study, pelvic fractures over an average of 9.2 days were also found to be well reduced by the CCRT. Thus, the time from injury to surgery was one of the references for CCRT; however, it was not an absolute indicator. CCRT should be performed before hematoma maturation and soft tissue fibrosis occur. Bedside skeletal traction of the lower extremities is highly recommended when definitive surgical treatment is not possible in the early phase.

### Experiences of CCRT

In our case, the countertraction method was flexible, adjustable, and could be manipulated, in addition to being labor saving. The correct patient position was crucial for CCRT procedures. To maintain the fluoroscopic position placement of the patient before traction, it was recommended to push the unaffected side of the lower limb to perform countertraction first and then perform traction of the injured side of the lower extremity. Note that the hip and knee should be extended during the countertraction push. To improve traction efficiency, adjusting the operating table to the position of low head and high foot was beneficial for strengthening the anti‐traction effect. In some cases, a small amount of hip flexion (10–30°) further improved the reduction. Fracture reduction usually requires external rotation of the affected hemipelvis and can be achieved either by manipulating the ilium or by using a Schanz pin placed in the iliac wing as a handle, as shown by Starr. In our series, the reduction quality of the hemipelvis in external rotation could be improved by internal rotation of the lower limbs when the reduction of the hemipelvis longitudinal displacement was achieved. Finally, the decision to use reduction and fixation methods was guided by patient‐specific factors, fracture type, and surgeon preference. In this study, the CCRT combined cannulated screw fixation of the posterior pelvic ring with anterior pelvic ring INFIX fixation, or external fixation. No CCRT related, or other major, complications occurred in our cases other than one case of wound fat liquefaction and one case of ossification.

It is important to obtain a high quality intraoperative imaging for successful reduction and fixation, and we need to identify confounding factors to avoid falling into the trap, such as patient body habitus, displaced fracture overlap and bowel gas.^29^ There is an unexpected case that the posterior pelvic ring was close to anatomical reduction during the performance of CCRT, free iliac fragments and anterior pelvic ring failed to achieve anatomical reduction. the surgical team decided not to make further adjustments after extensive communication with the patient and his family. Although the quality of reduction was only fair, his postoperative function was excellent, with a Majeed functional score of 100. The results of this study again verify that minimally invasive management and excellent reduction of the posterior pelvic ring is crucial for the recovery of function.^30^ Therefore, when performing CCRT, we should pay attention to the quality of the reduction of the posterior pelvic ring.

### Limitations

Nevertheless, this study has limitations. First, it is a single surgeon series and was only applied in 13 patients; further research into the effectiveness of the procedure using a randomized, controlled study with a larger sample size should be considered. Second, because of the individualized differences in pelvic injuries, our use of CCRT to reduce fractures relied on fluoroscopy and experience; therefore, traction force and direction cannot be quantified, which will lead to some difficulties in the technique being reproducible or widely applicable.

### Prospects of Clinical Application

As shown in this paper, the CCRT is a non‐invasive reduction technique with the characteristics of being economical and practical, easy to perform and effective in reducing tile C‐type pelvic injury. Thus, CCRT has promising application prospects in clinics. However, it is necessary to follow the indications and requires the surgeon to have reliable surgical techniques and fluoroscopy before clinical applications.

## Conclusion

In conclusion, the present study showed that CCRT is a non‐invasive closed reduction method for fresh Tile C pelvic fractures, and combined with the minimally invasive fixation can achieve a small surgical incision, decreased intraoperative bleeding, satisfactory fracture reduction, bony union, and function recovery. We believe that this study will help in the treatment of similar cases using fixation with closed reduction and minimally invasive surgery.

## Conflict of Interest Statement

The authors of this manuscript declare no relationships with any companies, whose products or services may be related to the subject matter of the article.

## Ethics Statement

Ethics approval and consent to participate: This project fully considered and protected the rights and interests of the study objects. It meets the criteria of the Ethical Review Committee. Ethical approval of this study was provided by the second affiliated hospital of Guangxi Medical University Review Board (No. 2022‐KY[0137]). Written informed consent for publication was obtained from all participants.

## Author Contributions

Wei Liu contributed to performed the operation and manuscript revising; Jianwen Cheng contributed to analysis and interpretation; Linke Huang, Chao Ning contributed to assist the operation, data collection; Jinmin Zhao and Feng Hu contributed to manuscript drafting and approval for publishing.

## Funding

Guangxi Natural Science Foundation of China, no. 2023GXNSFAA026320; The R&D project of Guangxi Zhuang Autonomous Region Key Trauma Surgery, no. GXKTS202203305; Guangxi Medical and Health Appropriate Technology development and application project, no. S2022094; Health Commission of Guangxi Autonomous Region self‐funded research project, no. Z‐A20230609.

## Data Availability

All data relevant to the study are included in the article or uploaded as supplementary information. All data relevant to the study are included in the article.
